# Mock Code: A Code Blue Scenario Requested by and Developed for Registered Nurses

**DOI:** 10.7759/cureus.938

**Published:** 2016-12-23

**Authors:** Kerry-Lynn Williams, Janice Rideout, Sherry Pritchett-Kelly, Melissa McDonald, Paula Mullins-Richards, Adam Dubrowski

**Affiliations:** 1 Faculty of Medicine, Memorial University of Newfoundland; 2 Clinical Learning and Simulation Centre, Memorial University of Newfoundland; 3 Eastern Health, Eastern Health; 4 Emergency Medicine, Pediatrics, Memorial University of Newfoundland

**Keywords:** simulation-based medical education, nursing simulation, nursing education

## Abstract

The use of simulation in medical training is quickly becoming more common, with applications in emergency, surgical, and nursing education. Recently, registered nurses working in surgical inpatient units requested a mock code simulation to practice skills, improve knowledge, and build self-confidence in a safe and controlled environment. A simulation scenario using a high-fidelity mannequin was developed and will be discussed herein.

## Introduction

Simulation-based medical education is an evolving field that allows trainees to practice skills, expand knowledge, and build self-confidence in a safe and controlled environment with no risk to patients [1]. Through the use of a high-fidelity mannequin simulator, participants can practice clinical skills, and the mannequin will respond in accordance with the students’ actions or inactions [2]. Recently, registered nurses working in surgery inpatient units expressed interest in participating in a simulation, in particular, a Code Blue scenario. The Quality, Patient Safety, and Risk Management Department of Eastern Health (EH) echoed staff concerns and approached the Clinical Learning and Simulation Centre (CLSC) for support. This technical report describes a unique collaboration between EH, a teaching hospital in St. John’s, and the CLSC, a simulation center based in Memorial University. After the EH nurse educator met with the CLSC staff, it was felt that practicing a code in a safe, simulated environment would be a means to improve provider confidence and ultimately improve patient safety. Targeted learning objectives for this scenario were developed, focusing on the recognition of the deteriorating patient, team approach to care, timely and appropriate Code Blue response initiation, effective patient care, and clear communication techniques. Therefore, this scenario was designed for registered nurses working in each of the surgery units at EH, including orthopedics, neurosurgery, urology, and general surgery/plastics/burns.

## Technical report

The simulation training session was conducted in the CLSC, Faculty of Medicine, Memorial University, using a high-fidelity mannequin simulator. This particular simulation used the Laerdal SimMan 3G™ (Laerdal, NY, USA). Prior to the session, a detailed scenario template was developed (Table 1) and submitted to the high-fidelity curriculum coordinator. The mannequin was then programmed, and the necessary tools for the simulation were developed and supplied.

**Table 1:** The detailed scenario template, which is submitted to the simulation lab’s technical staff, who then program the mannequin and supply necessary materials for the case.

Pre-Scenario		
You are a registered nurse working in a surgery inpatient unit. You are rounding on your patients.		
History		
Reason for admission	Sarah Pratt is a 59-year-old female electrician who underwent bilateral total knee replacements three days ago. She is alert and oriented to person, place, and time.	
Handover notes	She had her epidural topped and pulled yesterday and has had poor pain control since it was removed. Mrs. Pratt becomes very anxious when the pain medication starts to wear off and is refusing to ambulate with the physio. Dr. Blue assessed her and changed her pain medication to morphine 10 mg subcut Q4H PRN. Mrs. Pratt is receiving her Q4H medication regularly. She becomes nauseated with the pain medication and takes Gravol 50 mg intravenous (IV) each time with the morphine. Mrs. Pratt’s vitals have been stable. She is receiving O2 40% via venturi mask to keep her O2 sats at 92%. She is currently drowsy but easily aroused.	
Medications	Pariet – 20 mg PO daily Xarelto – 10 mg PO daily Acetaminophen – 650 mg PO QID Morphine – 2–5 mg subcut Q4H PRN	
Past medical history	Osteoarthritis, GERD, appendectomy (2000)	
Allergies	NKDA	
Social history	Smoker – 40 pack-year history	
Baseline vitals	HR 90 BP 100/60 spO2 92%, 40% venturi RR 18 T 38 eyes open, alert and talking, diaphoretic, bilateral crackles	
Begin Scenario: Learner enters the patient’s room		
Objective 1: Recognize the deterioration of the client’s condition and apply a team approach to care		
Additional data/findings	Vitals	Appropriate Learner Action
Client is sitting in bed, diaphoretic with O2 40% via venturi mask. About three minutes into the scenario, the client will state that she is not feeling well. She will feel weak and short of breath.	HR 110 BP 86/50 spO2 90% RR 30	Repeat vitals. Calls in other nurse(s) for help.
Objective 2: Initiate timely and appropriate Code Blue response		
Client arrests.	VF Arrest: HR VF RR 0	Nurse initiates Code Blue. Assigns team roles and sends someone to get the crash cart.
Objective 3: Provide effective client care in a Code Blue situation prior to and after the arrival of the resuscitation team		
Starts cardiopulmonary resuscitation (CPR).		Provide effective respirations with bag-valve-mask (BVM) ventilation, look for evidence of chest rising. Start compressions, minimum of two inches deep to a maximum of 1/3 depth of the chest. Bring crash cart to bedside and apply pads. Record events. Start timing for effective switch out of CPR.
Objective 4: Demonstrate clear effective communication techniques to health team members		
Resident arrives, first shock given.		Nurse gives situation, background, assessment, recommendation (SBAR). Resident will identify client needs shock.
Resident takes over team lead role and follows cardiac arrest algorithm.		Resume CPR.
Second shock given, orders epinephrine.		Epinephrine drawn up.
Epinephrine given.	Return of spontaneous circulation: HR 80 BP 80/50 spO2 88 RR 16	End of scenario.

To ensure a smooth experience for the learners, the team involved conducted a dry run of the scenario. Checklists based on the current guidelines [3-5] were developed and used during the scenario to provide an overall assessment of trainee competency (Table 2). Also, the mannequin was able to provide feedback on the quality of compressions (e.g., depth, rate) and ventilation (e.g., seal, rate, saturation). The high-fidelity curriculum coordinator and the high-fidelity educator at the CLSC, along with the nurse educator from EH, facilitated the scenario and participated in the subsequent debriefing.

**Table 2:** Mock code assessment form

Mock Code Assessment Form Team Lead: Participants: Scenario: Facilitator(s): Date:	
Critical Performance Steps	Check if correct
Recognizes client deterioration	
Assesses vital signs	
Performs respiratory assessment	
Recognizes need for help	
Assessment and Activation	
Checks for responsiveness. Shakes shoulder, shouts patient’s name, and scans chest for movement (5–10 seconds)	
Checks carotid pulse (minimum 5 seconds – maximum 10 seconds), if no pulse	
Calls Code Blue or directs another rescuer to activate a Code Blue by calling 2000 on the internal phone line	
Calls for help to bring crash cart or automated external defibrillator (AED) to bedside	
CPR Skills	
Place patient in supine position with backboard and begin CPR	
Proper hand placement (place on lower half of breast bone just above xiphoid process)	
Compression rate of at least 100 to 120 compressions per minute with a ratio of 30:2	
Adequate depth of at least 5 cm or 1/3 chest wall	
Allow the chest to recoil completely after each compression	
Changing compressors Q2 minutes or after five cycles of 30 compressions and two breaths	
Minimize interruptions in compressions	
Ventilation	
Inserts oropharyngeal or nasopharyngeal airway (if required)	
Positions mask properly on face	
Effectively ventilates with a BVM device giving each breath over one second with visible chest rise	
Gives proper ventilation rate and volume; gives two breaths after every 30 compressions	
Defibrillation	
Place pads correctly on chest without interruption in chest compressions	
Connects to defibrillator	
Turns on LifePak 20 (Physio Control, Washington, USA) and gets rhythm	
Clears before ‘analyze’ and ‘shock’	
Immediately resumes CPR after shock delivery	
Recognizes the two-minute mark to recheck pulse and rhythm	
Administers appropriate drug(s) and doses when ordered	
Team Leader	
Ensures high-quality CPR at all times	
Assigns team member roles (intravenous, airway, defibrillator, documentation, compressor)	
Looks for closed loop communication after directing tasks	
SBAR format report given	

Pre-briefing

A pre-briefing was held with all learners before the case. Learners were given a brief orientation to the simulation lab and the mannequin. The limitations of the simulation were reviewed, in particular, addressing technical issues with the mannequin and resource availability. The fiction contract – the agreement between the participants and instructors to proceed as if the simulation is real while simultaneously acknowledging that it is not – was readdressed [6]. Finally, trainees were advised that the case is strictly formative.

Case

This simulation scenario began with the patient in a surgical unit. She was sitting in bed, diaphoretic, with 40% O2 via venturi mask. The chart was placed on the overbed table for the nurse to review. About three minutes into the scenario, the patient stated that she was not feeling well and that she felt weak and short of breath. At this time, the nurse was expected to repeat the vitals. Sats dipped a little, the heart rate increased, and the blood pressure dropped. Then, in about 30 seconds, the patient arrested. The nurses participating had to initiate a Code Blue, provide effective respirations with BVM, start compressions, bring the crash cart to the bedside and apply pads, start timing for effective switch out of CPR, as well as record events. Once all the tasks were performed, the clinical educator entered the room portraying the role of the resident. The resident took over the team lead role and followed the cardiac arrest algorithm. Once the first dose of epinephrine was given, the client had a return of spontaneous circulation (ROSC).

Blood pressure and oxygen saturation readings were available when requested by the trainee. Visual feedback (looking for the chest to rise) therefore became important in this scenario.

A video (Video 1) showing a complete run-through of the scenario is included. It is intended to provide a step-by-step approach to the case.

**Video 1:** Mock code: a Code Blue scenario requested by and developed for RNs

Debriefing

Following the conclusion of the scenario, learners are provided with a formal debriefing. Care is taken during the debriefing to ensure that the number of debriefers is limited such that the debriefer-to-learner ratio does not exceed 1:1. The debriefing should be led by an experienced educator. The principles of good judgment and frame discovery should be central to the process. The points covered in the debriefing session are included in Table 3.

**Table 3:** Debriefing/guided reflection questions

How did you feel throughout the simulation experience?
What went well in this simulation?
What did not go well in this simulation?
Were you satisfied with your ability to work through the simulation?
If you were able to do it again, how would you have handled the situation differently?
Was there effective closed loop communication?
Do you think there was effective use of delegation? Why or why not?
Is there anything else you would like to discuss?

Post-scenario didactics

After the debriefing, a didactic session was held. During this session, instructors were able to address any learning needs, and trainees were given the opportunity to strengthen the knowledge gained through the simulation exercise. Points covered in the didactics included being aware of the proper procedure when you come upon a patient with cardiac arrest, the importance of effective communication during stressful situations, and describing staff actions as response. Learners were asked to identify ways to improve staff response and patient care during a Code Blue scenario. The mannequin used in the scenario was also programmed to be able to determine the effectiveness of the compressions and airway management, so that was discussed where appropriate. A video (Video 2) showing the correct way to draw up epinephrine was included for further learning.

**Video 2:** Instructional video showing how to draw up epinephrine

## Discussion

This scenario was designed to increase the comfort level of registered nurses in Code Blue situations. It has been shown that nurses—both recent graduates and highly skilled, experienced nurses—respond with anxiety during a code. This anxious response results in delays in initiating cardiopulmonary resuscitation, regardless of responder experience. Under these high-stress conditions, they were also observed to struggle with the equipment—the arrest cart, defibrillator, and BVM device. In some instances, it was noted that the backboard placement was not correct, compressions were not effective, and ventilations were not adequate [7]. High-fidelity nursing simulation, while it does not replace working with patients, provides the opportunity for participants to learn technical skills and build confidence with no risk to the patient [8].

This particular simulation was developed at the request of registered nurses working in surgery inpatient units. In this simulation, the key learning objectives for trainees include:

1. Recognizing the deterioration of the patient’s condition and applying a team approach to care; 2. Initiating timely and appropriate Code Blue response; 3. Providing effective patient care in a Code Blue situation prior to and after the arrival of the resuscitation team; 4. Demonstrating clear, effective communication techniques to health team members.

The case scenario was developed to allow the simulation to react according to the decisions made by trainees. Having an instructor complete the dry run both ensured that the scenario is of reasonable difficulty for the target population, and that it provided an opportunity for instructors to address any limitations of the scenario. Knowledge gaps, as well as any errors, were addressed in the debriefing and post-scenario didactic sessions.

This pilot collaboration between Eastern Health (EH), a teaching hospital, and the Clinical Learning and Simulation Centre (CLSC), Faculty of Medicine, Memorial University, has been very successful. Nursing staff and the Quality, Patient Safety, and Risk Management Department recognized the need for further training in this topic to increase the comfort level and knowledge of staff. The simulation training scenario has been built based on real needs observed in the hospital by the staff, developed collaboratively with the educators from the CLSC and EH, and delivered in the space using a shared funding model. Since completing the scenario, numerous positive evaluations have been received from participants. Also, some of those who have witnessed a code since participating in this scenario have stated that they were able to successfully put learned actions into practice. All in all, this has been a very successful pilot collaboration and a start to many other future endeavors.

## Conclusions

Code Blue situations can be very demanding and emotionally charged. Therefore, practice in a controlled environment can be beneficial for registered nurses, ultimately leading to improved patient outcomes. We have presented herein a mock Code Blue simulation, along with post-scenario didactics and teaching, designed for registered nurses working on surgical floors.
